# Outcomes of a Noninferiority Randomised Controlled Trial of Surgery for Men with Urodynamic Stress Incontinence After Prostate Surgery (MASTER)^☆^

**DOI:** 10.1016/j.eururo.2021.01.024

**Published:** 2021-06

**Authors:** Paul Abrams, Lynda D. Constable, David Cooper, Graeme MacLennan, Marcus J. Drake, Chris Harding, Anthony Mundy, Kirsty McCormack, Alison McDonald, John Norrie, Craig Ramsay, Rebecca Smith, Nikki Cotterill, Mary Kilonzo, Cathryn Glazener, on behalf of MASTER Trial Team

**Affiliations:** a Bristol Urological Institute, Southmead Hospital, North Bristol NHS Trust, Bristol, UK; b Health Services Research Unit (HSRU), University of Aberdeen, Aberdeen, UK; c School of Physiology, Pharmacology and Neuroscience, University of Bristol, Bristol, UK; d Department of Urology, Newcastle upon Tyne Hospitals NHS Foundation Trust, Newcastle, UK; e Translational and Clinical Research Institute, Newcastle University, Newcastle, UK; f Department of Urology, University College London Hospitals NHS Foundation Trust, London, UK; g Usher Institute, College of Medicine and Veterinary Medicine, University of Edinburgh, Edinburgh, UK; h Faculty of Health and Applied Science, University of West of England, Bristol, UK; i Health Economics Research Unit, University of Aberdeen, Aberdeen, UK

**Keywords:** Urodynamic stress incontinence, Male sling, Artificial urinary sphincter, Randomised controlled trial, Noninferiority

## Abstract

**Background:**

Stress urinary incontinence (SUI) is common after radical prostatectomy and likely to persist despite conservative treatment. The sling is an emerging operation for persistent SUI, but randomised controlled trial (RCT) comparison with the established artificial urinary sphincter (AUS) is lacking.

**Objective:**

To compare the outcomes of surgery in men with bothersome urodynamic SUI after prostate surgery.

**Design, setting, and participants:**

A noninferiority RCT was conducted among men with bothersome urodynamic SUI from 27 UK centres. Blinding was not possible due the surgeries.

**Intervention:**

Participants were randomly assigned (1:1) to the male transobturator sling (*n* = 190) or the AUS (*n* = 190) group.

**Outcome measurements and statistical analysis:**

The primary outcome was patient-reported SUI 12 mo after randomisation, collected from postal questionnaire using a composite outcome from two items in validated International Consultation on Incontinence Questionnaire-Urinary Incontinence Short Form questionnaire (ICIQ-UI SF). Noninferiority margin was 15%, thought to be of acceptable lower effectiveness, in return for reduced adverse events (AEs) and easier operation, for the sling. Secondary outcomes were operative and postoperative details, patient-reported measures, and AEs, up to 12 mo after surgery.

**Results and limitations:**

A total of 380 participants were included. At 12 mo after randomisation, incontinence rates were 134/154 (87.0%) for male sling versus 133/158 (84.2%) for AUS (difference 3.6% [95% confidence interval {CI} –11.6 to 4.6], *p*_NI_ = 0.003), showing noninferiority. Incontinence symptoms (ICIQ-UI SF) reduced from scores of 16.1 and 16.4 at baseline to 8.7 and 7.5 for male sling and AUS, respectively (mean difference 1.4 [95% CI 0.2–2.6], *p* =  0.02). Serious AEs (SAEs) were few: *n* = 6 and *n* = 13 for male sling and AUS (one man had three SAEs), respectively. Quality of life scores improved, and satisfaction was high in both groups. All other secondary outcomes that show statistically significant differences favour the AUS.

**Conclusions:**

Using a strict definition, urinary incontinence rates remained high, with no evidence of difference between male sling and AUS. Symptoms and quality of life improved significantly in both groups, and men were generally satisfied with both procedures. Overall, secondary and post hoc analyses were in favour of AUS.

**Patient summary:**

Urinary incontinence after prostatectomy has considerable effect on men’s quality of life. MASTER shows that if surgery is needed, both surgical options result in fewer symptoms and high satisfaction, despite most men not being completely dry. However, most other results indicate that men having an artificial urinary sphincter have better outcomes than those who have a sling.

## Take Home Message

The results of this large, high-quality trial allow surgeons to discuss and recommend the use of both the artificial sphincter and the sling procedure for postprostatectomy incontinence, as the results with respect to urine leakage were similar. However, men will wish to know that the majority will have some persistent urinary incontinence, although satisfaction with surgery was high: in the artificial urinary sphincter (AUS) group 91% and 85% were, respectively, completely and fairly satisfied, and 85% would recommend an AUS to a friend; and in the sling group, 72% were completely or fairly satisfied and 72% would recommend a sling to a friend. Similarly, most of the other secondary outcomes favoured the AUS over the sling procedure.

## 1 Introduction

Stress urinary incontinence (SUI) is a common symptom in men after radical prostatectomy and can be difficult to improve. Even after physiotherapy, 75% of men who remained incontinent after 6 wk have some leakage even 12 mo after their prostate surgery [1]. Implantation of the artificial urinary sphincter (AUS) is the recommended surgical procedure for men who have had insufficient benefit from conservative treatments, and still have troublesome SUI >12 mo after surgery [1], [2]. As the AUS is relatively expensive, requires specialist surgical skill to implant, and may require revisions over time, other surgical methods have been tested. These have not been evaluated in adequately powered randomised controlled trials (RCTs), as concluded in the 2011 Cochrane review and National Institute for Health and Clinical Excellence (NICE) guidance (CG97), with no new RCTs since these were published [1], [3], [4]. Male slings of many varieties have also been reported in case series over the past decade, and have been increasingly used for the treatment of postprostatectomy incontinence (PPI), again without high-level evidence [1], [2], [5]. The current NICE guidance (CG97), last updated in 2015 (accessed August 2019), remains unchanged [1]. The 2017 International Consultation on Incontinence (ICI) [2] states the following: the AUS is “the preferred treatment”; “male slings are an acceptable surgical approach with several years’ follow-up supporting their safety and efficacy in men with mild to moderate degrees of PPI”; and “injectable agents, even with repeated application, have a low success rate”. The 2018 European Association of Urology (EAU) guidelines are similar [5]. Hence, current authoritative recommendations support the need for an RCT such as MASTER, making its findings relevant to current clinical practice.

Owing to these uncertainties, the aim of MASTER was to compare the AUS and passive suburethral synthetic slings (“male slings”) in an RCT. At the planning stage of MASTER, there were little reasonable data on compressive slings (LE4 evidence), and compressive slings appeared to have higher complication and reoperation rates. Furthermore, the compressive sling technology was changing, and operative procedures appeared to have little standardisation. For these reasons, we decided that MASTER should reflect this and current practice, which was that the male slings used predominantly by the MASTER surgeons were passive in type (advanced). This decision is still supported by the current 2020 EAU guidelines, which recommend offering fixed slings, but add that “there is limited evidence that adjustable male slings can cure or improve SUI in men”, “there is limited evidence that early explantation rates are high”, and “there is no evidence that adjustability offers additional benefit over other types of sling”, each being LE3 evidence [6].

## 2 Patients and methods

The full methodology, including a flowchart, is described in the published protocol (https://trialsjournal.biomedcentral.com/articles/10.1186/s13063-018-2501-2) [7]. This report focusses on the 12-mo primary and main secondary outcomes. Future papers will report the 24-mo outcomes, and qualitative and economic outcomes.

### 2.1 Patients

Men who had decided, in discussion with their urologist, to have surgery for persistent bothersome SUI resulting from prostate surgery and were willing to be randomised between male sling and AUS were included in the MASTER RCT (International Randomised Controlled Trial Registry, ISRCTN49212975; registered on 22 July 2013). In line with current practice, men were not considered unless they had failed conservative treatment including pelvic floor exercises, stress incontinence was confirmed on urodynamics (UDS; urodynamic stress incontinence [USI]), and 12 mo had elapsed since their prostate surgery [7].

Men were excluded if they had had previous male sling or AUS surgery, had unresolved bladder neck contracture or urethral stricture after prostate surgery, had insufficient manual dexterity to operate the AUS device, or were unable to give informed consent or complete trial documentation [7].

### 2.2 Study design and ethical approval

MASTER was a multicentre, randomised, controlled, noninferiority trial of surgery for men with USI after prostate surgery. The primary outcome of the trial was a noninferiority comparison of the rates of incontinence at 12 mo after randomisation. Our reason for this approach was that if a male sling is inferior in the short term, then male slings will highly likely not be introduced throughout the NHS, irrespective of longer-term costs and consequences.

Participants were recruited from 27 UK urological centres. Ethical approval was received by the National Research Ethics Service (NRES) South West – Frenchay Research Ethics Committee: Reference Number 13/SW/0132, and all men gave written informed consent.

### 2.3 Randomisation and masking

Randomisation was carried out as close to the time of surgery as practical. Patients were randomised using a probabilistic algorithm to either a male sling or an AUS in a 1:1 ratio, minimising for type of prostate surgery (radical prostatectomy or transurethral resection of the prostate [TURP]), previous prostate radiotherapy (yes or no), and centre, using a remote automated computer-allocated randomisation system hosted at the Centre for Healthcare Randomised Trials (CHaRT) in Aberdeen, UK. Participants could not be blinded to their allocated procedure since the AUS requires the patient to operate the device, while the male sling does not.

### 2.4 Procedures

All surgeons were competent at AUS implantation. Surgeons who were relatively inexperienced in male sling implantation were mentored until deemed competent by principal investigators who were experienced in male sling surgery. Detailed guidance on surgical technique was written, for example, “balloon placed in extraperitoneal pocket”; the protocol was endorsed by all participating centres [7].

### 2.5 Clinical outcome and patient-reported outcome measures

The primary clinical outcome was any self-reported urinary incontinence (UI) at 12 mo after randomisation, a composite outcome derived from responses indicating any loss of urine to either of the two questions: “how often do you leak urine?” and “how much urine do you leak?” (from the validated International Consultation on Incontinence Questionnaire-Urinary Incontinence Short Form questionnaire [ICIQ-UI SF]) [8].

The data monitoring committee suggested using a less strict definition of the primary outcome; this included “less than once a week” and “a small amount” in the definitions of success and was analysed in the same way. This was taken to define only more severe cases as being incontinent at 12 mo, that is, those that leaked one or more times a week.

The secondary clinical outcome and patient-reported outcome measures (PROMs) were operative and postoperative details, adverse events (AEs), readmissions, UI (ICIQ-UI SF score [8]) and incontinence pad usage, 24-h pad test (weight of urine lost), and lower urinary tract symptoms (ICIQ-Male Lower Urinary Tract Symptoms [ICIQ-MLUTS]) [9]. Quality of life outcome measures included interference on everyday life (ICIQ-UI SF [8]), sexual matters (ICIQ-MLUTSsex [10]), general health measures (SF12 [11] and EQ-5D [12]), and satisfaction with treatment (ICIQ-satisfaction [13]). Men completed postal questionnaires and urinary diaries at 6 mo after surgery and 12 mo after randomisation, and had a clinical review and a 24-h pad test at 12 mo after surgery. Men who reported that they were dry at 12 mo did not need to complete the 24-h pad test if they did not wish to do so. Similarly, no 24-h pad test was available for those who did not attend clinic for their 12-mo review (in these cases, the clinic review occurred by telephone or from review of patient notes). Hence, quantification of incontinence severity in both cases was self-reported, categorised into small/moderate/large and semiobjective, using 24-h pad tests.

### 2.6 Sample size

Limited evidence from case series suggested that 20% of men would still be incontinent 12 mo after AUS. Assuming no difference between the arms, we required outcome data on 310 participants for 90% power to show that male slings were noninferior to AUS by a margin of 15%. The figure of 15% was a clinical decision on what noninferiority margin would be considered acceptable and was supported by the qualitative interviews with men (to be reported later). We allowed for a 15% loss to follow-up, inflating to 360 participants in total (180 per group) [7].

### 2.7 Statistical analysis

An analysis was done (by D.C.) based on randomised allocation (intention to treat), used data collected in the 12-mo follow-up questionnaire and the 12-mo clinic appointment. We used a generalised linear model with previous prostate radiotherapy and pad test weight at baseline and a random effect (intercept) for centre to analyse the primary outcome. We used the lower limit of the two-sided 95% confidence around the difference between groups to infer noninferiority between male slings and AUS, which is equivalent to a 2.5% one-sided type 1 error rate. We calculated a *p* value for observing the difference under the null hypothesis of inferiority.

EQ-5D [12] and ICIQ-UI SF [8] scores were analysed using a mixed-effect repeated-measure model on all available 6- and 12-mo data, adjusting for baseline outcome measure and design covariates (as for the primary outcome). Effect sizes are presented as the difference between groups with 95% confidence interval (CI) and a superiority *p* value. Differences between complication rates or readmission rates are presented as the risk difference and CI.

Pad use is analysed as a count variable with a negative binomial model adjusting for baseline pad use, previous radiotherapy, and clustering on centre. The frequency and amount of urine leakage are analysed using ordered logistic regression with the same design covariates as the primary outcome. These analyses were added to aid in the interpretation of the primary outcome.

A subgroup analysis of the primary outcome by baseline pad weight was performed as outlined in the statistical analysis plan. The subgroup analysis by UI diagnosis is post hoc as are both subgroup analyses of satisfaction with surgery.

## 3 Results

### 3.1 Participants

**Fig. 1:**
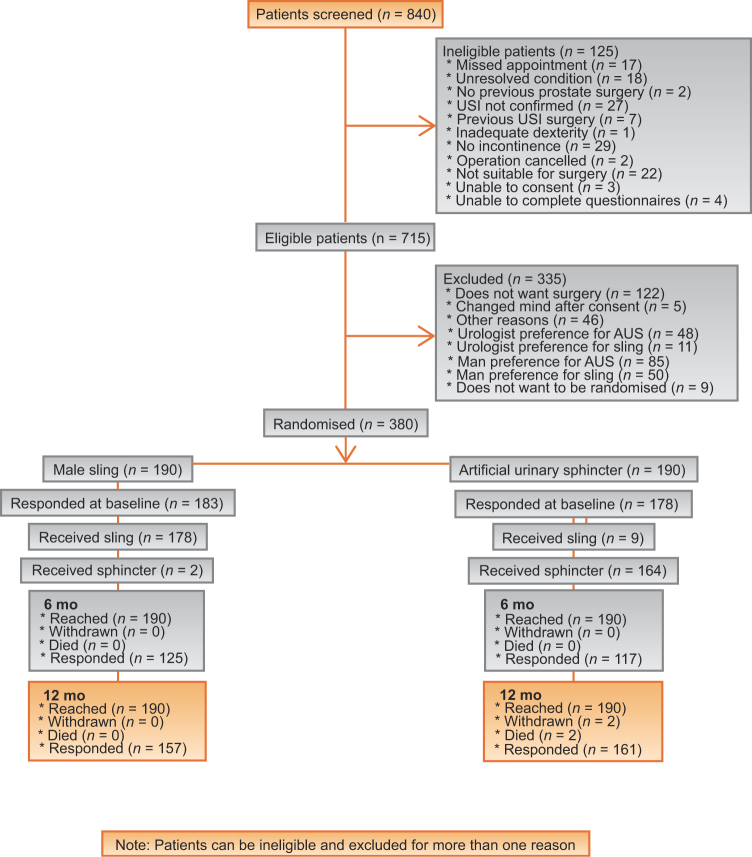
Trial profile. AUS = artificial urinary sphincter; USI = urodynamic stress incontinence.

Between 29 January 2014 and 28 December 2017, 380 men were randomised to receive a male sling or an AUS (190 in each group; Fig. 1). The groups were similar at baseline (Table 1).

**Table 1:** Baseline characteristics

	Male sling	AUS
	*N* = 190	*N* = 190
Age (yr)	68 (64, 71)	69 (63, 72)
ICIQ-UI SF	16 (14, 19); (*N* = 172)	17 (14, 19); (*N* = 166)
Score for effect on everyday life	8 (6, 10); (*N* = 178)	8 (7, 10); (*N* = 176)
24-h pad test result (g)	256 (89, 545); (*N* = 159)	267 (130, 554); (N = 159)
Pads used on an average day	3 (2, 4); (*N* = 180)	3 (2, 5); (*N* = 173)
EQ-5D	0.848 (0.760, 1.000); (*N* = 177)	0.850 (0.760, 1.000); (*N* = 172)
How often do you leak urine?		
Two or three times a week	3 (1.6)	1 (0.5)
About once a day	3 (1.6)	2 (1.1)
Several times a day	115 (60.5)	104 (54.7)
All the time	54 (28.4)	59 (31.1)
Missing	15 (7.9)	24 (12.6)
How much urine do you usually leak?		
A small amount	38 (20.0)	29 (15.3)
A moderate amount	76 (40.0)	84 (44.2)
A large amount	68 (35.8)	61 (32.1)
Missing	8 (4.2)	16 (8.4)
Do you wear pads or protection because of leaking urine?		
No	5 (2.6)	4 (2.1)
Yes	175 (92.1)	171 (90.0)
Missing	10 (5.3)	15 (7.9)
Received radiotherapy for prostatic disease	38 (20.0)	39 (20.5)
Leaking urine before first prostate operation		
No	174 (91.6%)	173 (91.1)
Yes	6 (3.2%)	10 (5.3)
Missing	10 (5.3%)	7 (3.7)
Reason for previous prostate surgery		
Prostate cancer	178 (93.7)	180 (94.7)
Benign prostate obstruction	8 (4.2)	9 (4.7)
Both	4 (2.1)	1 (0.5)
Previous surgery or treatments		
Radical prostatectomy	180 (94.7)	181 (95.3)
Channel TURP for obstructed prostate cancer	2 (1.1)	4 (2.1)
Transuretheral prostatectomy for benign prostatic obstruction	13 (6.8)	7 (3.7)
Retropubic prostatectomy for benign prostatic obstruction	1 (0.5)	–
Injectable treatment for stress urinary incontinence	9 (4.7)	8 (4.2)
Physiotherapy for stress urinary incontinence	94 (49.5)	83 (43.7)
Drug treatment with duloxetine for urinary stress incontinence	23 (12.1)	21 (11.1)
Drug treatment for other urinary/bladder problem	40 (21.1)	35 (18.4)
Any neurological disease	2 (1.1)	7 (3.7)

AUS = artificial urinary sphincter; ICIQ-UI SF = International Consultation on Incontinence Questionnaire-Urinary Incontinence Short Form questionnaire; TURP = transurethral resection of the prostate.

The summaries in this table are count and percentage for the categorical variables, and for continuous variables the summary is: median (lower quartile, upper quartile); (number of observations).

### 3.2 Primary outcome

There were 134/154 (87.0%) incontinent men in the male sling group versus 133/158 (84.2%) in the AUS group, 12 mo after randomisation (difference –3.6% [95% CI –11.6 to 4.6], *p*_NI_ = 0.003; Table 2), confirming noninferiority. The “less strict” definition of continence (66% for sling and 65% for AUS) also confirmed noninferiority (difference –1.7 [95% CI –10.1 to 6.7], *p*_NI_ = 0.001). The proportion of self-reported frequency of leakage was similar between the groups; however, the self-reported amount of leakage was higher in the male sling group (odds ratio 1.64 [95% CI 1.02–2.65], *p* =  0.04; Table 2). A subgroup analysis of the primary outcome is shown in the Supplementary material (Supplementary Table 4 and Supplementary Fig. 2A and 2B). All the subgroup analyses show a large amount of uncertainty around the effect sizes, but suggest that the male sling is inferior to the sphincter for men with greater incontinence at baseline (pad weight >250 g); however, the difference does not reach statistical significance. The subgroup analyses also suggest that the male sling may perform better than the AUS for men with pure SUI at baseline.

**Table 2:** Outcome data

	Male sling	AUS	Difference (95% CI); *p* value	Odds ratio (95% CI)
	*N* = 154	*N* = 158		
Incontinent a	134 (87.0)	133 (84.2)	–3.6 (–11.6, 4.6); *p*_NI_ = 0.003	0.75 (0.36, 1.54)
Incontinent—less strict b	102 (66.2)	103 (65.2)	–1.7 (–10.1, 6.7); p_NI_ = 0.001	0.92 (0.62, 1.35)
Per protocol	*N* = 151	*N* = 145		
Incontinent	131 (86.8)	123 (84.8)	–2.7 (–10.9, 5.6); *p*_NI_ = 0.002	0.80 (0.39, 1.63)
Incontinent (less strict)	99 (65.6)	95 (65.5)	–1.2(–9.7, 7.3); *p*_NI_ = 0.001	0.95 (0.64, 1.39)
	*N* = 151	*N* = 154	Odds ratio (95% CI) c	
How often do you leak urine?				
Never	21 (13.9)	25 (16.2)	1.25 (0.89, 1.76); *p* = 0.2	
Once a week or less	31 (20.5)	27 (17.5)		
Two or three times a week	13 (8.6)	13 (8.4)		
About once a day	12 (7.9)	20 (13.0)		
Several times a day	59 (39.1)	60 (39.0)		
All the time	15 (9.9)	9 (5.8)		
	*N* = 154	*N* = 158		
How much urine do you usually leak?				
None	20 (13.0)	26 (16.5)	1.64 (1.02, 2.65); *p* = 0.04	
A small amount	91 (59.1)	103 (65.2)		
A moderate amount	29 (18.8)	21 (13.3)		
A large amount	14 (9.1)	8 (5.1)		
			Effect size (95% CI) d	
ICIQ-UI SF	8.7 (6.1); (*N* = 151)	7.5 (5.3); (*N* = 153)	1.4 (0.2, 2.6); *p* = 0.02	
Score for effect on everyday life	3.5 (3.4); (*N* = 155)	2.7 (2.9); (*N* = 157)	0.9 (0.2, 1.5); *p* = 0.01	
EQ-5D	0.809 (0.260); (*N* = 151)	0.813 (0.274); (*N* = 158)	–0.019 (–0.062, 0.024); *p* = 0.4	
Wears pads or other protection	102/148 (68.9%)	102/150 (68.0%)	2.1 (–11.0, 15.2); *p* = 0.8	
Pads used in an average day	1.6 (2.1); (*N* = 146)	1.3 (1.7); (*N* = 150)	1.25 (0.99, 1.58); *p* = 0.06	
Pad weight (g)	30.0 (85.3); (*N* = 50)	73.7 (451.6); (*N* = 44)		

AUS = artificial urinary sphincter; CI = confidence interval; ICIQ-UI SF = International Consultation on Incontinence Questionnaire-Urinary Incontinence Short Form questionnaire.

Cells are *n*/*N* (%); the *p* value is from a test of noninferiority.

a The definition of incontinent is a participant who has indicated any frequency other than “never” or an amount greater than “none”.

b The less strict definition of incontinent is a participant who has indicated a frequency greater than “once a week or less” or an amount greater than “a small amount”.

c The reference group is AUS.

d The effect sizes for ICIQ-UI SF score for effect on everyday life and EQ-5D are adjusted mean differences. For “wearing pads or other protection”, the effect size is an adjusted risk difference, and for “pads used in an average day”, it is an incidence rate ratio adjusted for baseline pad quantity and previous radiotherapy, and clustered by centre.

### 3.3 Secondary outcomes

In both groups, there was a reduction from baseline to 12 mo in the ICIQ-UI SF score [8], which measures incontinence symptoms. The effect size shows incontinence symptoms, at 12 mo, to be worse in the male sling group with the CI excluding zero and therefore indicating that this difference is significant (mean difference 1.4 [95% CI 0.2–2.6], *p* =  0.02). Preoperative incontinence was analysed by symptomatic type, SUI, and urgency urinary incontinence (UUI), and by severity of incontinence, with five grades from “never” to “all the time”. At baseline, 16% of men had pure SUI, 61% had predominant SUI, 12% had equal severity SUI and UUI, 4% had predominant UUI, and 7% did not enter their severity grades. At 12 mo, the men with pure SUI had better dry rates (21% and 48%) on the two outcome measures than those with SUI, plus any element of UUI (13% and 32%). The odds ratios for the original definition of incontinence is 0.42 (95% CI 0.20, 0.88; *p* =  0.02), and for the less strict definition of incontinence it is 0.42 (95% CI 0.19, 0.93; *p* =  0.03). Both of these show that men with pure SUI are less likely to be incontinent at 12 mo.

In response to the question: “Overall, how much does leaking urine interfere with your everyday life?” (where zero indicates “not at all” and 10 “a great deal”), the difference of 0.9 (95% CI 0.2–1.5, *p* =  0.01) shows that those in the male sling arm experience significantly greater interference in their everyday lives (Table 2, and Supplementary Fig. 1A and 1B).

Postoperative pad use is similar in both the sling and the AUS group. The effect size (incidence rate ratio) of 1.20 suggests a slightly higher use in the sling group, but the CI of (0.95, 1.53) shows that the level of uncertainty makes the difference not significant (Table 2, and Supplementary Fig. 1C and 1D).

#### 3.3.1 Satisfaction

**Fig. 2:**
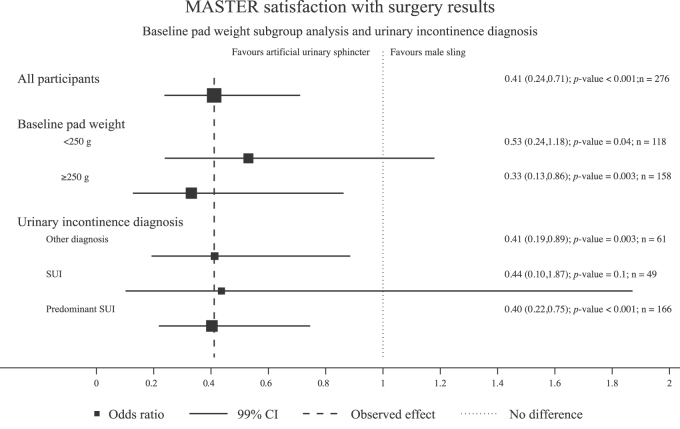
Forest plot of satisfaction with surgery. CI = confidence interval; SUI = stress urinary incontinence.

Satisfaction (ICIQ-satisfaction [13]) with the outcome of surgery was much greater for the AUS group: 104 (72.2%) were completely or fairly satisfied) in the male sling group and 125 (90.6%) in the AUS group (difference –18.4% [–27.2, –9.6], *p* <  0.001; Supplementary Table 1); 72.0% and 84.5% of men in the sling and AUS groups, respectively, would recommend their procedure to a friend (difference –12.5% [–21.7, –3.2], *p* =  0.01; Supplementary Table 2); satisfaction was positively associated with a greater decrease in self-reported degree of urine loss (*p* <  0.001); and for men with a preoperative 24-h pad weight of <250 g, 72% of the sling and 88% of the AUS men were satisfied, and in men with a pad weight of >250 g, satisfaction rates were 77% in the sling and 95% in the AUS group. An odds ratio of 2.17 (95% CI 0.43, 11.02, *p* =  0.4; Fig. 2) shows that when the type of incontinence (pure SUI, predominant SUI, SUI = UUI, and predominant UUI) is analysed according to baseline 24-h pad tests (<250 or >250 g) and satisfaction with surgery, there is greater satisfaction in the AUS group than in the male sling group, irrespective of the degree of leakage (odds ratio 0.41 [95% CI 0.24, 0.71], *p* <  0.001).

#### 3.3.2 Surgery and AEs

Details of surgery received are shown in Figure 1. The mean durations of the surgery and hospital stay were, respectively, 1.7 h and 1.6 d for the male sling and 2.1 h and 1.5 d for the AUS group. In-patient AEs (expected and nonexpected) were recorded during the men’s hospital stay (Table 3). There were 225 nonserious AEs in 154/180 (85.6%) men in the male sling group compared with 189 in 147/173 (85.0%) men in the AUS group. A post hoc analysis shows that significantly more men in the male sling group (*n* = 49, 27.2%) had two or more AEs than those in the AUS group (*n* = 28, 16.2%; difference = 11.0%, 95% CI 2.5%, 19.5%; *p* =  0.01).

**Table 3:** Adverse events

	Male sling	AUS
Received operation	180/190(94.7)	173/190(91.1)
Serious adverse events a	6	13
Total number adverse events	225	189
	*N* = 180	*N* = 173
Number of participants with any adverse events	152 (84.4)	147 (85.0)
Number of adverse events per participant		
0	28 (15.6)	26 (15.0)
1	102 (56.7)	119 (68.8)
2	34 (18.9)	18 (10.4)
3	11 (6.1)	6 (3.5)
≥4	5 (2.8)	4 (2.3)
Type of adverse event		
Postop catheter required	28 (15.6)	8 (4.6)
Catheter required for >24 h	20 (11.1)	6 (3.5)
Urinary tract infection		2 (1.2)
Pyrexia	1 (0.6)	3 (1.7)
Wound infection	3 (1.7)	1 (0.6)
Sepsis, septicaemia, or abscess	1 (0.6)	
Retention requiring surgery	1 (0.6)	
Bowel obstruction		1 (0.6)
Constipation	1 (0.6)	3 (1.7)
New urinary tract symptoms	2 (1.1)	
Tape or male sling complications	1 (0.6)	
Device exposure/extrusion requiring no treatment		1 (0.6) b
Acute or chronic pain	1 (0.6)	1 (0.6)
Oral pain relief given	139 (77.2)	137 (79.2)
Parenteral pain relief given	13 (7.2)	12 (6.9)
Antibiotic treatment for postop infection	6 (3.3)	10 (5.8)
Other adverse events^c^	4 (2.4)	2 (1.2)

	Male synthetic sling, no radiotherapy	Male synthetic sling, previous radiotherapy	Artificial urinary sphincter, no radiotherapy	Artificial urinary sphincter, previous radiotherapy
Received operation	144/152 (94.7%)	36/38 (94.7%)	139/151 (92.1%)	34/39 (87.2%)
Serious adverse events	3	3	9	4
Total number of adverse events	187	38	154	35
	*N* = 152	*N* = 38	*N* = 151	*N* = 39
Number of participants with any adverse events	124 (86.1%)	28 (77.8%)	121 (87.1%)	26 (76.5%)
Number of complications per participant				
0	20 (13.9%)	8 (22.2%)	18 (12.9%)	8 (23.5%)
1	82 (56.9%)	20 (55.6%)	98 (70.5%)	21 (61.8%)
2	28 (19.4%)	6 (16.7%)	15 (10.8%)	3 (8.8%)
3	9 (6.3%)	2 (5.6%)	6 (4.3%)	
≥4	5 (3.5%)		2 (1.4%)	2 (5.9%)
Type of adverse event				
Postop catheter required	24 (16.7%)	4 (11.1%)	7 (5.0%)	1 (2.9%)
Catheter required for >24 h	14 (9.7%)	6 (16.7%)	6 (4.3%)	
Urinary tract infection			1 (0.7%)	1 (2.9%)
Pyrexia	1 (0.7%)		2 (1.4%)	1 (2.9%)
Wound infection	3 (2.1%)		1 (0.7%)	
Sepsis, septicaemia, or abscess	1 (0.7%)			
Retention requiring surgery	1 (0.7%)			
Bowel obstruction			1 (0.7%)	
Constipation	1 (0.7%)		2 (1.4%)	1 (2.9%)
New urinary tract symptoms	2 (1.4%)			
Tape or sling complications	1 (0.7%)			
Device exposure/extrusion requiring no treatment			1 (0.7%)	
Acute or chronic pain	1 (0.7%)			1 (2.9%)
Oral pain relief given	114 (79.2%)	25 (69.4%)	112 (80.6%)	25 (73.5%)
Parenteral pain relief given	12 (8.3%)	1 (2.8%)	8 (5.8%)	4 (11.8%)
Antibiotic treatment for postop infection	5 (3.5%)	1 (2.8%)	10 (7.2%)	
Other adverse events	3 (2.1%)	1 (2.8%)	2 (1.4%)	

AUS = artificial urinary sphincter; SAE = serious adverse event.

a SAEs were as follows: male sling group—recatheterisation requiring or prolonging hospital stay (*n* = 3), mesh erosion (*n* = 1), infection (urosepsis, *n* = 1), and development of coffee ground vomit (*n* = 1); AUS group—recatheterisation requiring or prolonging hospital stay (*n* = 3), infection (*n* = 3), erosion of device (*n* = 2), haematoma (*n* = 1), bruising and inflammation (*n* = 1), anaesthetic complication (*n* = 1), urinary retention/voiding difficulties (*n* = 1), pain (*n* = 1), transient hypotension (*n* = 1), and thrombosis (*n* = 1), and three SAEs in one man.

b Herniated AUS reservoir through abdominal hernia (device still present, not causing any problems).

c Other adverse events: the four events in the male sling group are as follows: extended hospital stay due to few minutes of loss of transient consciousness, left leg pain, and difficulty bearing weight; large postvoid residuals; retention requiring recatheterisation, and rash in the right groin; the two events in the AUS group are urinary retention after discharge and intraoperative ventricular tachycardia.

Serious adverse events (SAEs) were few, and experienced by six men in the male sling group (recatheterisation requiring or prolonging hospital stay [*n* = 3], mesh erosion [*n* = 1], infection [urosepsis, *n* = 1], and development of coffee ground vomit [*n* = 1]) and 11 men in the AUS group (recatheterisation requiring or prolonging hospital stay [*n* = 3], infection [*n* = 3], erosion of device [*n* = 2], haematoma [*n* = 1], bruising and inflammation [*n* = 1], urinary retention/voiding difficulties [*n* = 1], pain [*n* = 1], transient hypotension [*n* = 1], and thrombosis [*n* = 1], and three SAEs in one man). Men who receive a sling are less likely to experience an SAE, but there is a considerable level of uncertainty around the estimate (incidence rate ratio 0.44 [0.16, 1.25], = 0.1).

The only difference in AEs was a greater number of catheter problems in the male sling group: 45 men (25.0%) in the male sling group versus 13 men (7.5%) in the AUS group (difference 17.5% [10.0, 24.9], *p* ≤  0.001).

#### 3.3.3 Patient-reported AEs

AEs identified from the 12-mo questionnaire referred to the 12 mo from randomisation (Supplementary Table 3). There were 51/157 (32.5%, male sling) and 36/161 (22.2%, AUS) AEs reported, with higher numbers in the male sling group for new bladder symptoms (7.6% in the male sling vs 1.2% in the AUS group; difference 6.4% [1.9, 10.9], *p* =  0.01), infections including urinary tract infections (12.1% in the male sling vs 6.8% in the AUS group; difference 4.6% [–1.5, 10.7], *p* =  0.1), and surgical site pain (21.7% in the male sling vs 11.2% in the AUS group; difference 10.5% [2.4, 18.6], *p* =  0.01). Device problems (not working as well as hoped) were more frequent with AUS (7.6% in the male sling vs 10.6% in the AUS group; difference –2.9% [–9.2, 3.4], *p* =  0.4).

#### 3.3.4 Readmissions for additional surgery within 12 mo

There were 16 readmissions for further or additional UI surgery, 13 in the male sling group and three in the AUS group. A total of 13 male slings failed to improve the men’s incontinence, 12 men in the male sling group had an AUS implanted, and one man received a second sling—none of these were removed. In the AUS group, three had revision for mechanical failure (change of balloon reservoir, balloon replacement, or repositioning; 5.5% [1.2, 9.7], *p* =  0.01; Table 4).

**Table 4:** Readmissions within 12 mo

	Male sling	Artificial urinary sphincter
	*N* = 180	*N* = 173
Further USI surgery	13 (7.2%)	2 (1.2%)
Additional surgery		1 (0.6%)
Participants with any readmission	13 (7.2%)	3 (1.8%)
Pain due to further surgery		1 (0.6%)

USI = urodynamic stress incontinence.

Overall, 70 (20%) of the 353 randomised men who received surgery had previous radiotherapy. In those readmitted, 53% had had radiotherapy (7/14 from the male sling group and 3/5 from the AUS group), while only 18% (60/334) of those not readmitted received prior radiotherapy, confirming that prior radiotherapy leads to worse outcomes from both treatments (34.7% [11.8, 57.5], *p* =  0.0002).

## 4 Discussion

The primary outcome showed that AUS and male sling surgeries were equally clinically effective at 12 mo, confirming noninferiority. However, MASTER found a much higher prevalence of incontinence after PPI surgery than is reported in the existing literature [14], [15], [16], [17], with only 15% of men in both groups saying that they never leaked. The 2018 American Urological Association guidelines [18] give the “cure” rate for a male sling at 62%, but point out that the definition of cure varies between 14 papers, as are the definitions of “improved”: little evidence was found for the AUS. There are several possible reasons for our finding of a low cure rate. Firstly, our use of a very strict definition of “no incontinence” using a PROM to determine the primary outcome ensured stringent criteria for cure. Secondly, this finding is perhaps not surprising, as present PPI surgical techniques cannot be expected to fully recreate the continence mechanisms that existed prior to radical prostatectomy. Thirdly, the wide inclusion criteria applied in MASTER resulted in our cohort exhibiting the full spectrum of UI severity at study entry. Despite the large proportion of men still having some degree of UI after implantation of an AUS or a sling for PPI, incontinence is much improved in most men. From clinical experience, we know that men after PPI surgery are often satisfied with their surgery even if not completely dry. With this in mind, a satisfaction outcome measure was included in MASTER. As discussed below, this instrument has allowed us to understand how we can present the results of MASTER to men with PPI who are contemplating surgery.

Both the primary and several secondary outcome measures are dependent on PROMs. We felt that patients’ statements as to whether or not they were dry, and on other aspects of their experience after surgery, should be the most important sources of data for interventions that are primarily designed to improve their quality of life. Not only is MASTER the first high-quality RCT to examine outcomes after surgical treatment of PPI by comparison of the two most commonly used procedures, the AUS and the male sling, but it is also by far the largest study within this subject area and the first to use a full range of validated PROMs to evaluate outcomes, making it generalisable across current practice.

Some might regard pad testing or UDS as more objective than PROMs, and therefore a better choice when selecting a primary outcome. However, pad testing has a complex literature, with some recommending that a 1-h pad test should include a standardised exercise regime; these issues make pad testing problematic for the primary outcome. We felt that the 24-h test was superior and a useful metric at baseline, but we also knew from experience that it would probably be hard to get a lot of men to repeat their 24-h pad tests at 12 mo if they were more or less dry, and the research nurses confirmed this to be so. Similarly, we did not feel that UDS should be an outcome measure. As UDS is the standard of care in the UK prior to PPI surgery, we felt that it should be included in MASTER. It was our view that, if SUI was not demonstrated at UDS, then other causes, such as detrusor overactivity, might be the cause of the man’s symptoms and he would not respond to PPI surgery. In MASTER, we wished to be sure that all men had the condition that we sought to evaluate, namely, SUI. We intend to analyse and publish separately any symptom associations with preoperative urodynamic features, such as detrusor overactivity, which might have impacted outcome.

By using a PROM, it was possible to analyse outcomes by incontinence subgroups and severity of incontinence, and this indicated that men with pure SUI had better dry rates (21% and 48%, respectively), on the two outcome measures, than those presenting with SUI plus any element of UUI (13% and 32%, respectively). These data are supported by the satisfaction data related to baseline pad weight and incontinence group, which favours AUS over male slings (Fig. 2). This information will help in a more complete discussion prior to PPI surgery.

Whist the primary outcome showed that a male sling is not inferior to an AUS, and UI (using the validated ICIQ-UI SF [8]) was greatly reduced after surgery in both groups, differences were seen in the secondary outcome measures, almost all of which show greater improvement for the AUS than for the male sling group. The additional quality of life question showed that improvement favoured the AUS, the self-reported amount of urine leakage postoperatively was higher in the male sling group, and the mean pad usage decreased more in the AUS group. There was also higher overall satisfaction with an AUS, despite the majority of men still reporting some urine leakage: this included 85% versus 72% in the sling group, who were prepared to recommend the AUS operation to a friend. Men in both groups were generally satisfied with their improvement and accepted the fact that, although not cured, they were sufficiently improved to be satisfied.

There were fewer AEs and lower rates of further surgery in the men having an AUS than in men having a male sling, although there were more SAEs for the AUS group. A total of 225 AEs were reported in the male sling group compared with 189 in the AUS group, with many more catheter-related AEs in the male sling group. It is likely that this was because some men were not routinely catheterised after male sling surgery and required catheterisation within 24 h after surgery due to inability to void adequately. At 12 mo after surgery, significantly more repeat continence surgeries were necessary in the male sling group than in the AUS group, largely due to the failure of slings to control leakage in men. However, it is likely that sling failures will occur early, and it may be that the 5-yr follow-up, which we intend to perform, will show later failures in the AUS men. Of all men, from both groups, who required additional surgery, radiotherapy was over-represented, with 53% of these men having had radiotherapy prior to radical prostatectomy. More men in the male sling group reported problems with infections, new bladder symptoms, and pain at the site of surgery or elsewhere. Although we do not feel that chronic pain is a major problem for men after PPI surgery, because of the controversies in women over mesh used for stress incontinence and pelvic organ prolapse surgery, we are planning an early, more detailed review of pain in our patient group. On the contrary, men in the AUS group had more problems with the device, as might be expected for a device that requires patient activation, and few SAEs were experienced by six men in the male sling group compared with 13 SAEs experienced by 11 men in the AUS group—neither of these outcomes reached statistical difference.

The often-stated potential advantages of a male sling compared with an AUS are reduction in length of hospital stay, lower cost, fewer complications, and fewer reoperation rates. However, none of these were confirmed in MASTER. The length of stay for sling surgery was not reduced, and this may have been because most men were catheterised, as some surgeons may have felt it wise to catheterise all men after sling surgery for a minimum of 24 h. However, there is a theoretical “trade-off” between fewer urinary tract infections if the men do not have a postop catheter and a faster discharge home, but with the need for a percentage requiring recatheterisation after surgery, as was seen in the sling group. In addition, more male sling patients required repeat continence surgery. The suggested potential of male slings to reduce the number of complications and reoperation rates of the AUS was not apparent at 12 mo, and longer follow-up will be required to assess whether there are significant differences in this respect.

As device-related complications do not always occur in the first 12 mo, longer-term data acquisition has been advised for all surgical devices, as highlighted recently by the Royal College of Surgeons of England [19]; hence, we will seek 5-yr follow-up of our established MASTER trial cohort. The planned 24-mo follow-up will be important for its health-economic analyses (HEAs), which will add cost-effectiveness data to longer-term clinical effectiveness to guide overall decision-making on service design and provision. The HEAs will be comprehensive and include, for example, the use of medications and reoperations and other relevant admissions to hospital.

Prior to MASTER, the widespread view was that a trial comparing these interventions would not be possible due to a lack of equipoise from both surgeons and patients. Despite this, we recruited above the minimum target sample size. We think that the success in recruitment was in part due to the surgeons from the sites meeting early in the planning stage. We tackled the issue of our equipoise and challenged each other’s views, for example, that male slings were for mild and AUS for severe incontinence, and whether men who had had radiotherapy should be excluded. We agreed that the evidence on these issues was usually of level 4 and considered that the literature did not contain sufficient evidence to be able to write the inclusion and exclusion criteria for many of the views we had held. Hence, we felt that the RCT should be pragmatic and not exclude individuals unless there were clear safety issues or convincing data on poor outcome. We have used a multicentre, pragmatic trial design, with wide inclusion criteria, to ensure that both procedures were tested in a manner aligned with present clinical practice, making the results generalisable. We were able to test many of the hypotheses that can be found in literature dominated by case series, which can provide only lower-level quality of evidence [4]. Hence, there is now good-quality evidence from the PROMS to show that even men with greater leakage can be offered the sling procedure, after full discussion of the findings of MASTER, as most men were satisfied with a sling. When we tested for a difference between the <250 g and >250 g leakage levels, on the baseline 24-h pad tests, the effect size was 0.62 (0.16, 2.36; *p* =  0.4). However, in a requested post hoc analysis of the question “leak urine when sleeping?”, there was a statistical association between increased leakage during sleep and higher 24-h pad weights, for example, for leakage “occasionally”, the mean pad weight was 327 ml, whereas for “most of the time”, it was 615 ml. Although men who had developed UI after TURP for benign prostatic obstruction, prior to their PPI surgery, were included, there were only eight of 190 in the male sling group and nine of 190 in the AUS group, and these numbers were too small to offer a useful analysis.

A limitation of this study is that we were unable to compare the severity of the MASTER men’s incontinence with that of men in the case series reports, because the data from previous non-RCT studies do not contain adequate information for comparison. However, men in MASTER were more incontinent than the men included in the only other reported RCT [3], which contained a significant number of men described as “minimally incontinent”.

The wide range of PROMs allowed us to look at the trial results from a number of other perspectives. Additionally, a recent study by Machioka et al [15] supports our use of the validated ICIQ-UI SF as an effective tool in evaluating UI after radical prostatectomy. The use of PROMs for the primary and secondary outcomes at baseline and 12 mo, together with the large number of men included, means that results from MASTER can be regarded as authoritative. MASTER provides a significant advance to the literature, and is the highest-quality evidence available to date to guide patients and clinicians when considering surgical intervention for PPI.

In summary, the results of MASTER indicate that men should be counselled that both AUS and male slings are effective in significantly reducing urinary leakage. In the majority of men, both procedures improve quality of life, and satisfaction rates are high. However, “true cure”, which is the absence of any leakage under any circumstances, cannot be expected in most men. A man debating on whether to have a male sling or an AUS is likely to decide on the basis of the benefits and risks of the two procedures. MASTER shows that the advantage of the simplicity of use of a male sling (in that it does not require the man to manipulate his device in order to void) must be balanced against some increased risks. The male sling group had higher incidences of postoperative recatheterisation, and at 12 mo, the male sling patients reported larger leakage quantities and higher use of pads, with less improvements in quality of life and satisfaction. The male sling group also has higher incidences of infections, new bladder symptoms, and surgical site pain. However, patients report a higher incidence of device problems (fault with balloon reservoir or requiring repositioning) with an AUS. Finally, patients should be told that in the male sling group, within 12 mo, there is a three-fold higher risk of needing a repeat continence operation and an increased risk of pain. All statistical secondary and post hoc analyses were in favour of the AUS. As to why there should be advantages of the AUS over the sling, we can speculate that it is possible that the circumferential positive pressure of the AUS works somewhat better than the unidirectional elevation offered by the sling. In addition, we are able to state that previous radiotherapy increases the risk of reoperation in any operation for PPI.

There is little evidence to support the view that a male sling should be used for mild/moderate incontinence and an AUS for severe incontinence. Both devices perform well irrespective of whether or not the baseline 24-h pad testing showed leakage above or below 250 g (Supplementary Table 4).

These initial results allow a better-informed consent process when a man discusses PPI procedures with his surgeon.

## 5 Conclusions

MASTER has provided the highest evidence (level 1) at 12 mo that both the AUS and the male sling procedure lead to improvement in most men with PPI, but do not completely “cure” the majority. The trial outcomes can be used when advising men on whether to have PPI surgery and whether they should consider the AUS or male sling procedure or neither. The results will allow detailed discussion regarding the benefits and risks of both procedures; the better results of the AUS group have to be balanced with the need to manually operate the AUS, while the male sling group has higher complication rates. These results have provided, for the first time, information that allows fully informed consent. The evidence from MASTER will also inform clinical practice through evidence-based guidelines.

  ***Author Contributions*:** Lynda D. Constable had full access to all the data in the study and takes responsibility for the integrity of the data and the accuracy of the data analysis.

  *Study concept and design*: Abrams, MacLennan, Drake, Mundy, McCormack, McDonald, Norrie, Ramsay, Smith, Cotterill, Kilonzo, Glazener.

*Acquisition of data*: Abrams, Constable, independent research sites (principal investigators listed in Acknowledgements).

*Analysis and interpretation of data*: Cooper analysed the data with oversight from MacLennan, which were interpreted by all authors.

*Drafting of the manuscript*: Abrams, Constable, and Cooper wrote the first draft of the manuscript which was reviewed, modified, and approved by all other authors.

Critical revision of the manuscript for important intellectual content: All authors.

*Statistical analysis*: Cooper, MacLennan.

*Obtaining funding*: Abrams, MacLennan, Drake, Mundy, McCormack, McDonald, Norrie, Ramsay, Smith, Cotterill, Kilonzo, Glazener.

*Administrative, technical, or material support*: Abrams, MacLennan, McDonald, Constable, Harding, Drake, Cooper, Norrie.

*Supervision*: Abrams, MacLennan, McDonald.

*Other*: None.

***Financial disclosures*:** Lynda D. Constable certifies that all conflicts of interest, including specific financial interests and relationships and affiliations relevant to the subject matter or materials discussed in the manuscript (eg, employment/affiliation, grants or funding, consultancies, honoraria, stock ownership or options, expert testimony, royalties, or patents filed, received, or pending), are the following: Paul Abrams reports grants, personal fees and other from Astellas, and personal fees from Pfizer, Sun Pharma, Ipsen, Pierre Fabre, and Coloplast, outside of submitted work. Chris Harding reports personal fees from Astellas, Pfizer, Ferring, Allergan, and Medtronic, other from Pierre Fabre, AMS/Boston, Astellas, and Medtronic; and grants from NIHR HTA Grant, outside the submitted work. Marcus J. Drake reports personal fees and nonfinancial support from Astellas, and personal fees from Asofarma and Ferring, outside of submitted work.

  ***Funding/Support and role of the sponsor*:** The UK National Institute for Health Research Health Technology Assessment (NIHR HTA) programme (project number 11/106/01) is the funder of the study and approved the study proposal, but had no role in the study design, data collection, data analysis, data interpretation, or writing of the report.

  ***Data sharing*:** All data requests should be submitted to the corresponding author for consideration. Access to anonymised data may be granted following review.

  ***Acknowledgements***: The authors wish to thank the men who participated in MASTER. We also thank Fiona Cherry, Lana Mitchell, Georgia Mannion-Krase, Maria Ntessalen, Andrea Fraser, and Dianne Dejean for their secretarial support and data management; Kirsty Shearer, Caroline Peet, Karen Campbell, and Elerita Flammini (previous trial managers/assistant trial managers); the programming team in CHaRT, led by Gladys McPherson (to 2016) and Mark Forrest (2016–present); members of the PMG for their on-going support and advice; independent members of the TSC and DMC; site principal investigators (PIs); and the staff at the recruiting sites (listed in the Supplementary material) who facilitated recruitment, treatment, and follow-up of trial participants. The MASTER PIs were as follows: Hashim Hashim (PI; Southmead Hospital, Bristol), Nikesh Thiruchelvam (PI; Addenbrookes Hospital, Cambridge), Yz Almallah (PI, Queen Elizabeth Hospital, Birmingham), Jas Rai (PI; Leicester General Hospital, Leicester), Chris Harding (PI; Freeman Hospital, Newcastle),Jeremy Ockrim (PI; Westmoreland Street Hospital, UCLH), Sheilagh Reid (PI; Royal Hallamshire Hospital, Sheffield), Simon Fulford (PI; James Cook Hospital, Middlesbrough), Richard Parkinson (PI; Nottingham University Hospital, Nottingham), Suzie Venn (PI; St Richard’s Hospital, Chichester), Graeme Conn (PI; Southern General Hospital, Glasgow), Rowland Rees (PI; Southampton General Hospital, Southampton), Melissa Davies (Salisbury District Hospital, Salisbury), Neil Harris (PI; St James University Hospital, Leeds), Andrew Sinclair (PI; Stepping Hill Hospital, Stockport), Arun Sahai (PI; Guys Hospital, Guy’s-St Thomas), Tina Rashid (PI; Charing Cross Hospital, Imperial), Roland Morley (PI), Laurence Clarke (PI; Salford Royal Foundation Trust, Salford); John Bolton (PI; Bradford Royal Infirmary, Bradford), Amar Alhasso (PI; Western General Hospital, Edinburgh), Alun Thomas (PI; Royal Gwent Hospital, Newport), Talal Jabbar (PI), Davendra Sharma (PI; St George’s Healthcare NHS Trust, St George’s), James Moore (PI; Eastbourne District General Hospital, Eastbourne), Ian Beckley (PI; Pinderfields Hospital, Wakefield), Christian Seipp (PI; Wrexham Maelor Hospital, Wrexham), Liaqat Chowoo (PI; Bedford Hospital NHS Trust, Bedford), Charlotte Foley (PI; Lister Hospital, Stevenage), Andrew Baird (PI; University Hospital Aintree, Liverpool). The views and opinions expressed herein are those of the authors and do not necessarily reflect those of the UK Health Technology Assessment Programme, the National Institute of Health Research, the National Health Service, or the Department of Health. Independent members of the (1) Trial Steering Group and (2) Data Monitoring Committee who oversaw this study were as follows: (1) Howard Kynaston (chair), Neville Goodman (Consumer Representative), Christopher Walker (Patient representative), Suzanne Hagen, and Tom McNicholas, and (2) Jonathan Cook (Chair), John Parry, and Mark Speakman. We would like to thank previous collaborators of the MASTER trial: Charles Boachie, Robert Pickard, and Gladys McPherson.
