# Supplementary material for: Outcomes of a Noninferiority Randomised Controlled Trial of Surgery for Men with Urodynamic Stress Incontinence After Prostate Surgery (MASTER)[image]
Source: Eur Urol. 2021 Jun;79(6):812–23. doi: 10.1016/j.eururo.2021.01.024 (PMC8175331; doi:10.1016/j.eururo.2021.01.024)
Supplement: Supplementary file 1 [file mmc1.docx]

**Supplementary material**

**Outcomes of a non-inferiority randomised controlled trial of surgery for men with urodynamic stress incontinence after prostate surgery (MASTER)**

**Contents**

[**Supplementary Figure 1A – Interfere with everyday life at Baseline** 2](#_Toc61366866)

[**Supplementary Figure 1B – Interfere with everyday life at 12M** 2](#_Toc61366867)

[**Supplementary Figure 1C – Pad use at Baseline** 3](#_Toc61366868)

[**Supplementary Figure 1D – Pad use at 12 Months** 3](#_Toc61366869)

[**Supplementary Figure 2 – Subgroup analysis forest plot of urinary incontinence at 12 Months** 4](#_Toc61366870)

[**Supplementary Table 1 Satisfaction with treatment at 12 months^1^** 5](#_Toc61366871)

[**Supplementary Table 2 Recommend surgery to a friend** 5](#_Toc61366872)

[**Supplementary Table 3 Self-reported problems at 12 months** 5](#_Toc61366873)

[**Supplementary Table 4a** Subgroup analysis of Urinary incontinence in men with a pad loss < 250ml based on original primary outcome definition 6](#_Toc61366874)

[**Supplementary Table 4b** Subgroup analysis of Urinary incontinence in men with a pad loss 250ml or more original strict primary outcome definition 6](#_Toc61366875)

[**Supplementary Table 4c** Subgroup analysis of Urinary incontinence in men with a pad loss < 250ml based on less strict definition of incontinence 6](#_Toc61366876)

[**Supplementary Table 4d** Subgroup analysis of Urinary incontinence in men with a pad loss of 250ml or more based on less strict definition of incontinence 6](#_Toc61366877)

[**Acknowledgements** 8](#_Toc61366878)

# **Supplementary Figure 1A – Interfere with everyday life at Baseline**

Participant responses to the question “Overall, how much does leaking urine interfere with your everyday life?” (where zero is “not at all” to 10, “a great deal”) at baseline, in both the male sling and the AUS groups.

# **Supplementary Figure 1B – Interfere with everyday life at 12M**

Participant responses to the question “Overall, how much does leaking urine interfere with your everyday life?” (where zero is “not at all” to 10, “a great deal”) 12 months after randomization, in both the male sling and the AUS groups

# **Supplementary Figure 1C – Pad use at Baseline**

Participant report of the total number of pads/pantiliners worn on an average day (24 hours), in both the male sling and the AUS groups at baseline.

# **Supplementary Figure 1D – Pad use at 12 Months**

Participant report of the total number of pads/pantiliners worn on an average day (24 hours), in both the male sling and the AUS groups 12 months after randomization.

# **Supplementary Figure 2 – Subgroup analysis forest plot of urinary incontinence at 12 Months**

# **Supplementary Table 1 Satisfaction with treatment at 12 months^1^**

|  | **Male Synthetic Sling** | **Artificial Urinary Sphincter** | **Total** |
| --- | --- | --- | --- |
| Completely satisfied | 58(40.3%) | 79(57.3%) | 137(48.6%) |
| Fairly satisfied | 46(31.9%) | 46(33.3%) | 92(32.6%) |
| Fairly dissatisfied | 17(11.8%) | 8(5.8%) | 25(8.9%) |
| Very dissatisfied | 19(13.2%) | 3(2.2%) | 22(7.8%) |
| Not sure | 4(2.8%) | 2(1.5%) | 6(2.1%) |
| Not answered^2^ | 46(24.2%) | 52(27.4%) | 98(25.8%) |
| Total | 190 | 190 | 380 |

Pearson chi2(4) = 18.6   P-value = 0.001^3^

^1^Raw data for anyone who has responded to the question ‘Overall how satisfied are you with the results of your surgery for urine leakage?’ regardless of whether they received surgery. ^2^Not answered is all of those randomised to the two interventions. ^3^The chi-square test is only on those who have responded to the satisfaction question.

# **Supplementary Table 2 Recommend surgery to a friend**

|  | **Male Synthetic Sling** | **Artificial Urinary Sphincter** | **Total** |
| --- | --- | --- | --- |
| Yes | 108(72.0) | 123(81.5) | 231(76.7) |
| No | 29(19.3) | 6(4.0) | 35(11.6) |
| Not answered | 13(8.7) | 22(14.5) | 35(11.6) |
| Total | 150 | 151 | 301 |

Pearson chi2(2) = 18.4 P-value < 0.001

Participant response to the question ‘Would you recommend this surgery to a friend?’ at 12 months after randomization.

# **Supplementary Table 3 Self-reported problems at 12 months**

|  | **Male Synthetic Sling** | **Artificial Urinary Sphincter** |
| --- | --- | --- |
|  |  |  |
| 12-month questionnaire completed | 157/190(82.6%) | 161/190(84.7%) |
|  |  |  |
| Bowel obstruction | 2/157(1.3%) | 2/161(1.2%) |
| Constipation | 18/157(11.5%) | 21/161(13.0%) |
| New bladder or urinary symptoms | 12/157(7.6%) | 2/161(1.2%) |
| Urinary tract infection | 15/157(9.6%) | 9/161(5.6%) |
| Other infection | 4/157(2.5%) | 2/161(1.2%) |
| Device problems | 12/157(7.6%) | 17/161(10.6%) |
| Sexual problem | 18/157(11.5%) | 20/161(12.4%) |
| Wound breakdown | 2/157(1.3%) | 2/161(1.2%) |
| Pain at site of surgery | 34/157(21.7%) | 18/161(11.2%) |

**Supplementary Tables 4 Subgroup analysis - Testing for a difference in the effect of a sling on incontinence between the two pad loss groups**

# **Supplementary Table 4a** Subgroup analysis of Urinary incontinence in men with a pad loss < 250ml based on original primary outcome definition

|  | **Male Synthetic Sling** | **Artificial Urinary Sphincter** | | **Total** |
| --- | --- | --- | --- | --- |
| Not incontinent | 10(12.7) | 11(14.5) | 21(13.5) | |
| Incontinent | 58(73.4) | 55(72.4) | 113(72.9) | |
| Missing | 11(13.9) | 10(13.1) | 21(13.5) | |
| Total | 79(100.0) | 76(100.0) | 155(100.0) | |

# **Supplementary Table 4b** Subgroup analysis of Urinary incontinence in men with a pad loss 250ml or more original strict primary outcome definition

|  | **Male Synthetic Sling** | **Artificial Urinary Sphincter** | **Total** |
| --- | --- | --- | --- |
| Not incontinent | 8(10.0) | 12(14.4) | 20(12.3) |
| Incontinent | 55(68.8) | 57(68.7) | 112(68.7) |
| Missing | 17(21.2) | 14(16.9) | 31((19.0) |
| Total | 80(100.0) | 83(100.0) | 163(100.0) |

# **Supplementary Table 4c** Subgroup analysis of Urinary incontinence in men with a pad loss < 250ml based on less strict definition of incontinence

|  | **Male Synthetic Sling** | **Artificial Urinary Sphincter** | **Total** |
| --- | --- | --- | --- |
| Not incontinent | 26(32.9) | 24(31.6) | 50(32.3) |
| Incontinent | 42(53.2) | 42(55.3) | 84(54.2) |
| Missing | 11(13.9) | 10(13.1) | 21(13.5) |
| Total | 79(100.0) | 76(100.0) | 155(100.0) |

# **Supplementary Table 4d** Subgroup analysis of Urinary incontinence in men with a pad loss of 250ml or more based on less strict definition of incontinence

|  | **Male Synthetic Sling** | **Artificial Urinary Sphincter** | **Total** |
| --- | --- | --- | --- |
| Not incontinent | 18(22.5) | 24(28.9) | 42(25.8) |
| Incontinent | 45(56.3) | 45(54.2) | 90(55.2) |
| Missing | 17(21.2) | 14(16.9) | 31(19.0) |
| Total | 80(100.0) | 83(100.00) | 163(100.0) |

**Supplementary Table 5** Adverse Events split by randomised group and previous radiotherapy treatment

|  | Male Synthetic Sling  no radiotherapy | Male Synthetic Sling previous radiotherapy | Artificial Urinary Sphincter no radiotherapy | Artificial Urinary Sphincter previous radiotherapy |
| --- | --- | --- | --- | --- |
| Received operation | 144/152(94.7%) | 36/38(94.7%) | 139/151(92.1%) | 34/39(87.2%) |
| **Serious Adverse Events^1^** | 3 | 3 | 9 | 4 |
| Total number of adverse events | 187 | 38 | 154 | 35 |
|  | N=152 | N=38 | N=151 | N=39 |
| Number of participants with any adverse events | 124 (86.1%) | 28 (77.8%) | 121 (87.1%) | 26 (76.5%) |
| **Number of complications per participant** |  |  |  |  |
| 0 | 20 (13.9%) | 8 (22.2%) | 18 (12.9%) | 8 (23.5%) |
| 1 | 82 (56.9%) | 20 (55.6%) | 98 (70.5%) | 21 (61.8%) |
| 2 | 28 (19.4%) | 6 (16.7%) | 15 (10.8%) | 3 (8.8%) |
| 3 | 9 (6.3%) | 2 (5.6%) | 6 (4.3%) |  |
| 4 or more | 5 (3.5%) |  | 2 (1.4%) | 2 (5.9%) |
| **Type of Adverse Event** |  |  |  |  |
| Post op catheter required | 24 (16.7%) | 4 (11.1%) | 7 (5.0%) | 1 (2.9%) |
| Catheter required for > 24 hours | 14 (9.7%) | 6 (16.7%) | 6 (4.3%) |  |
| Urinary tract infection |  |  | 1 (0.7%) | 1 (2.9%) |
| Pyrexia | 1 (0.7%) |  | 2 (1.4%) | 1 (2.9%) |
| Wound infection | 3 (2.1%) |  | 1 (0.7%) |  |
| Sepsis, septicaemia or abscess | 1 (0.7%) |  |  |  |
| Retention requiring surgery | 1 (0.7%) |  |  |  |
| Bowel obstruction |  |  | 1 (0.7%) |  |
| Constipation | 1 (0.7%) |  | 2 (1.4%) | 1 (2.9%) |
| New urinary tract symptoms | 2 (1.4%) |  |  |  |
| Tape or sling complications | 1 (0.7%) |  |  |  |
| Device exposure/extrusion requiring no treatment |  |  | 1 (0.7%) |  |
| Acute or chronic pain | 1 (0.7%) |  |  | 1 (2.9%) |
| Oral pain relief given | 114 (79.2%) | 25 (69.4%) | 112 (80.6%) | 25 (73.5%) |
| Parenteral pain relief given | 12 (8.3%) | 1 (2.8%) | 8 (5.8%) | 4 (11.8%) |
| Antibiotic treatment for post-op infection | 5 (3.5%) | 1 (2.8%) | 10 (7.2%) |  |
| Other Adverse Events^2^ | 3 (2.1%) | 1 (2.8%) | 2 (1.4%) |  |

^1^SAEs were: male sling group (re-catheterisation requiring or prolonging hospital stay (n=3), mesh erosion (n=1), infection (urosepsis, n=1), developed coffee ground vomit (n=1)), AUS group ((re-catheterisation requiring or prolonging hospital stay (n=3), infection (n=3), erosion of device (n=2), haematoma (n=1), bruising and inflammation (n=1), anaesthetic complication (n=1), urinary retention/voiding difficulties (n=1), pain (n=1), transient hypotension (n=1), thrombosis (n=1), one man had three SAEs). ^2^Other adverse events: The four events in the male sling group are: Extended hospital stay due to few minutes loss of transient consciousness and left leg pain and difficulty bearing weight, large post-void residuals, patient went into retention and was re-catheterised, rash in right groin. The two events in the AUS group are urinary retention post discharge and intraoperative ventricular tachycardia. ^2^Herniated AUS reservoir through abdominal hernia (device still patent, not causing any problems).

# **Acknowledgements**

**Other staff members in the clinical centres were as follows:**

**Bristol**, Southmead Hospital: Debbie Delgado, Constance Shiridzinomwa, Markus Drake, Helena Burden, Kate Warren, Julie Plant, Leigh Morrison, Katie McDonald, Carol Brain, Lyndsey, Johnson; **Cambridge**, Addenbrookes Hospital: Kelly Leonard, Lisa Geoghegan, Suzanne Biers; **Birmingham**, Queen Elizabeth Hospital: Mohammed Belal , Yuko Smith, Alice Longe, Annette Nilsson, Carol Green, Daniella Lynch, Martin Joinson; **Leicester**, Leicester General Hospital: Tim Terry, Tina Rashid, Jackie Parker, Robert Radcliffe; **Newcastle**, Freeman Hospital: Peter Murphy, Wendy Robson, Nicola Brown, Victoria Lavin, Rachel Forrest, Bernadette Kilbane, Paul Hindmarch, Lynn.D Langhorne; **UCLH**, Westmoreland Street Hospital: Anthony Mundy, Daniella Andrich, Tamsin Greenwell, Rizwan Hamid, Anastasia Frost, Mariya Dragova, Mahreen Pakrad; **Sheffield**, Royal Hallamshire Hospital: Susannah Hulton, Alison Hyde, Anne Frost, Nadir Osman, Chris Hillary; **Middlesbrough**, James Cook Hospital: Julie Potts, Mary Gartwaite, Julie McGivern, Clare Proctor, Alycon Walker, Karoline Middleton, Martyn Cain, Matthew Bunting, Kerry Hebbron; **Nottingham**, Nottingham University Hospital: Andy Jarvis, Bria McAllister; **Chichester**, St Richard’s Hospital: Yolanda Baird, Isobel Amey, Alison Misselbrook, Sally Moore, Susanna Greenslade, Marian Flynn-Batham, Matthew Smith, Julie Wheatley; **Glasgow**, Southern General hospital: Simon Morton, Alastair McKay; **Southampton**, Southampton General Hospital: McDonald Mupudzi, Elisabeth Harouzet, Miranda Kean, Andrew Guy, Emma Levell, Meirion Ford, Michelle Smith, Caroline Andrews, Robbie Speigal, Abbie Morley, Margarida Salgueiro, Clare Hutchison, Winington Ruiz, Sanchia Triggs, Kimberley Harris; **Salisbury**, Salisbury district Hospital: Melissa Davies, Katie Chadwick, Ruth Fennelly, Caroline Clarke, Alpha Anthony, Eve Parker, Michele Tribbeck, Kate Ames; **Leeds**, St James University Hospital: Lorraine Wiseman (nee Lamb); **Stockport**, Stepping Hill hospital: Helen Cochrane, Sarah Scanlon, Lara Smith, Julie Grindey, Nnaemeka Eli, Alissa Kent, Sarah Connolly, Patricia Clitheroe, Patricia Coughlan; **Guy’s- St Thomas**, Guys Hospital: Nigel Butter, Jai Seth, Claire Taylor, Angel Garcia-Imhor, Sachin Malde, Matthew Hogben, Nicholas Favre Walker, Temitope Bankole, Naomi Hare, Samantha Broadhead; **Imperial**, Charing Cross Hospital: Matthias Winkler, Sanela Andrijac, Gillian Hornzee, Fatima Akbar, Daisy Floyd; **Salford**, Salford Royal Foundation Trust: Melanie Taylor, Kieran O’Flynn, Zoe Swann, Vicky Thomas; **Bradford**, Bradford Royal Infirmary: Richard Benton, Linda Bamford, Helen Robertshaw, Hayley Inman, Anne Kay, Agapios Grentzis, Jane Sewell, Kelvin Stewart, Kay Cockroft; **Edinburgh**, Western General Hospital: Emma Whitecross, Shiobhan McCaskey, Charlotte Smith, Laurence Stewart, Emma Fleming, Rachel Campbell, Katherine Laurence; **Newport**, Royal Gwent Hospital: Sarah Scourfield, Coral Seymour; **St George’s**, St George’s Healthcare NHS Trust: Agne Sekmokaite, Ruth Millett, Ella Foncel, Marios Hadjipavlou, Abigail Seward; **Eastbourne**, Eastbourne District General Hospital: Emma Edmunds, Anne Cowley, Penny Whithing, Jenni Law, Jackie Terry, Kelly Mintrim; **Wakefield**, Pinderfields Hospital: Victoria Dean, Beverley Taylor, Jim Anderson, Stephen Littler, Julie Ball, Aimee Hayton-Bolt, Asa Ali, Janine Heeley; **Wrexham**, Wrexham Maelor Hospital: Stacy Ackerley, Linzi Williams, Kelly Andrews, Lisa Ashley, Rachel Davies, Emma Hall, Natalie Hughes, Paula Conway, Mary Roberts, Jane Stockport, Claire Watkins, Jackie Morris, Rachel Hughes; **Bedford**, Bedford Hospital NHS Trust: Graham Smith, Charina Smith, Ann Britchford, Beena David, Retno Wulandari; **Stevenage**, Lister Hospital: Linda Fowler, Clare Collins, Louise Peacock, Jemma Gilmore, Faith Wilson; **Liverpool**, University Hospital Aintree: Jordan Ewing, Melanie Morrison, Marc Lucky.
